# Supplementary material for: Screening of Candidate Genes Associated with Brown Stripe Resistance in Sugarcane via BSR-seq Analysis
Source: Int J Mol Sci. 2022 Dec 7;23(24):15500. doi: 10.3390/ijms232415500 (PMC9778799; doi:10.3390/ijms232415500)
Supplement: Supplementary file 1 [file ijms-23-15500-s001.zip › Supplementary_Material - Figure S1.pdf]

International Journal of
Molecular Sciences

Supplementary Material

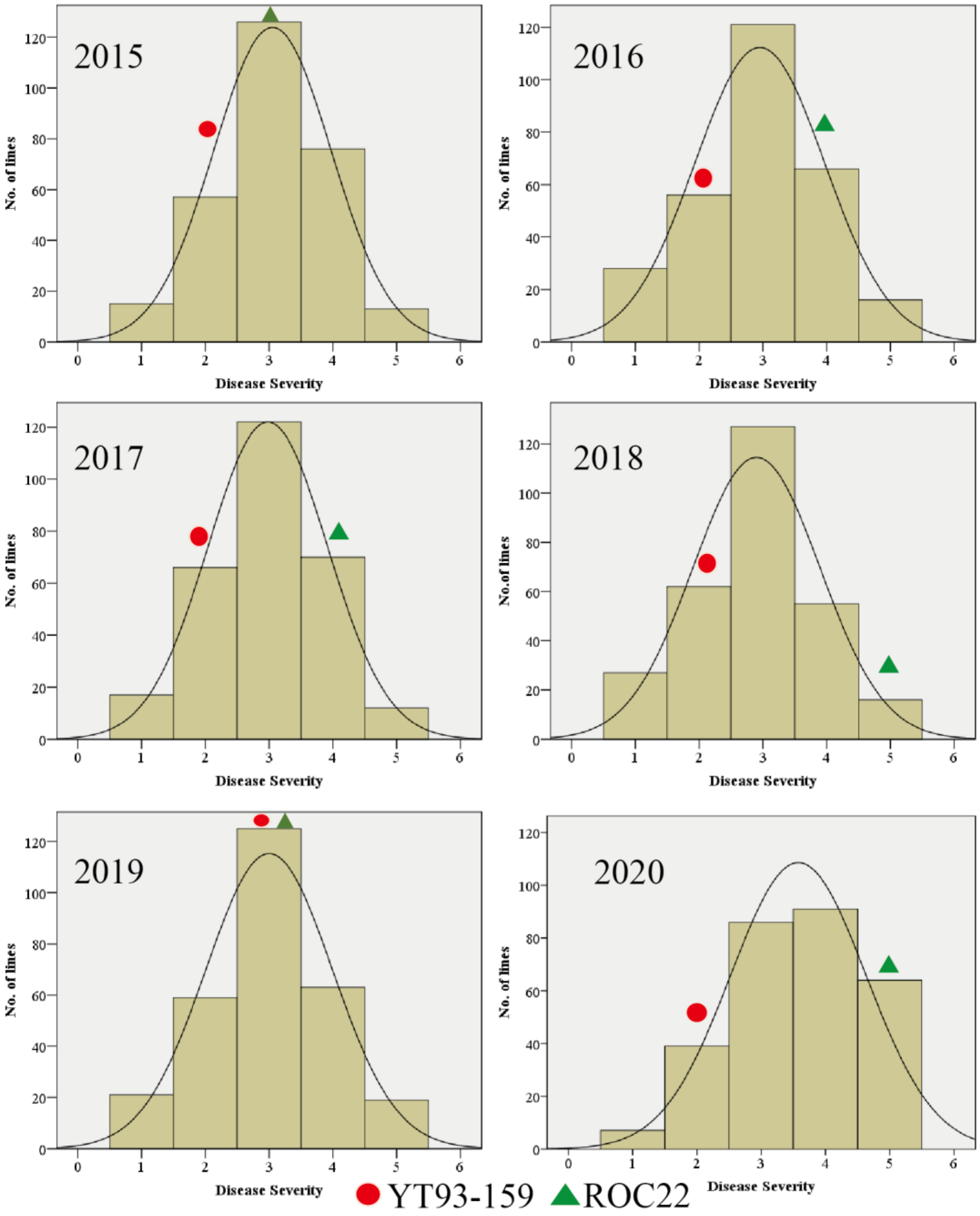

Figure S1. Frequency distribution for disease severity of SBS among the 287 F_1 individuals from the F_1 segregating population under six habitats.
