# Supplementary material for: Screening of Candidate Genes Associated with Brown Stripe Resistance in Sugarcane via BSR-seq Analysis
Source: Int J Mol Sci. 2022 Dec 7;23(24):15500. doi: 10.3390/ijms232415500 (PMC9778799; doi:10.3390/ijms232415500)
Supplement: Supplementary file 1 [file ijms-23-15500-s001.zip › Supplementary_Material - Figure S3.pdf]

International Journal of
Molecular Sciences

Supplementary Material

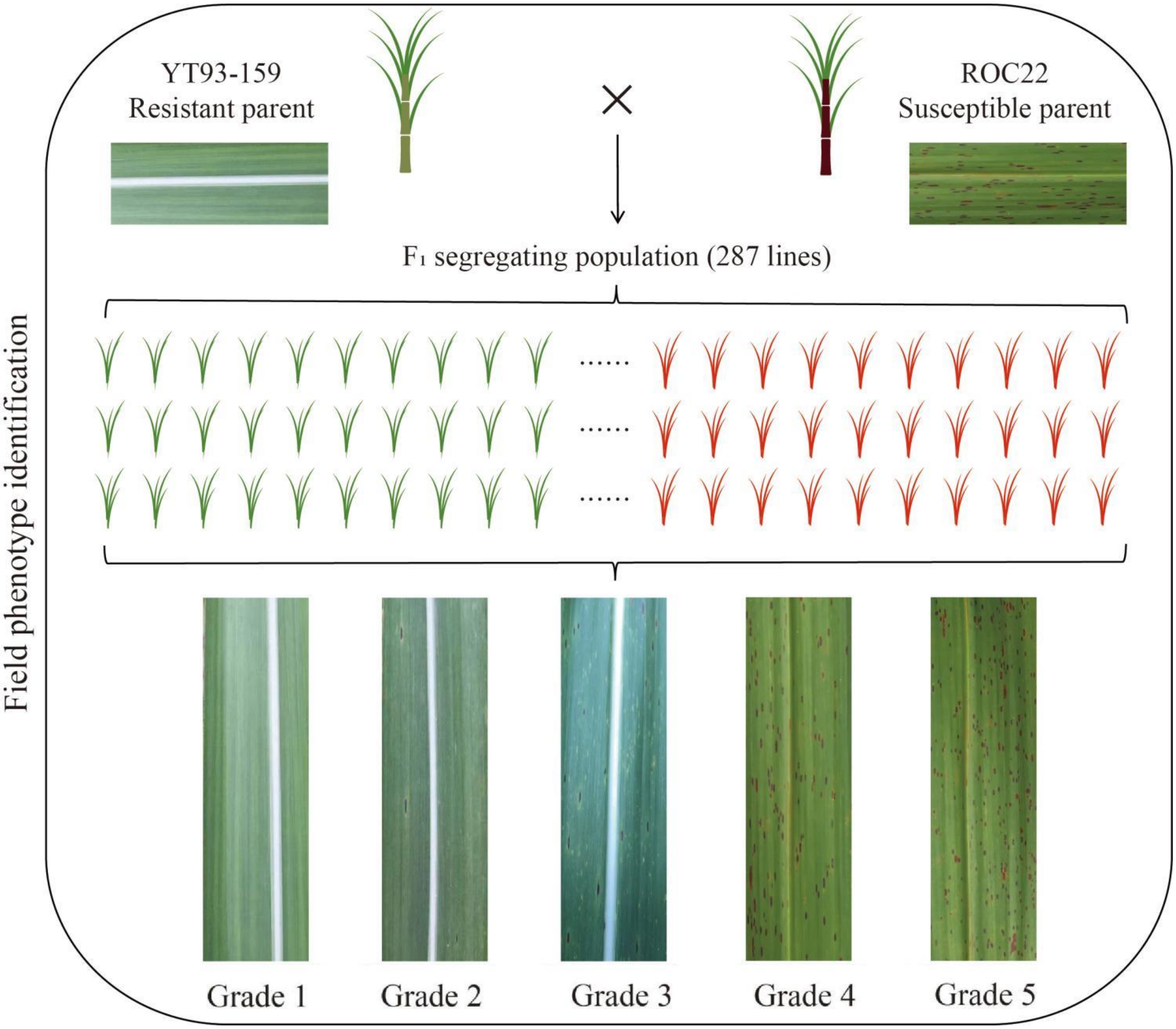

Figure S3. Symptom on sugarcane healthy leaves or the leaves infected by sugarcane brown stripe disease in two parents and their F_1 segregating individuals.
