# Supplementary material for: Screening of Candidate Genes Associated with Brown Stripe Resistance in Sugarcane via BSR-seq Analysis
Source: Int J Mol Sci. 2022 Dec 7;23(24):15500. doi: 10.3390/ijms232415500 (PMC9778799; doi:10.3390/ijms232415500)
Supplement: Supplementary file 1 [file ijms-23-15500-s001.zip › Supplementary_Material - Table S1.pdf]

International Journal of
Molecular Sciences

Supplementary Material

Table S1 The information of sugarcane brown stripe disease severity for 30 progenies were selected for construction of the resistant- or susceptible- mixed bulks and two parents in 2020

Line ID[a]	SBS[b]-susceptible mixed bulks			Line ID	SBS-resistant mixed bulks		
	Repeat I	Repeat II	Repeat III		Repeat I	Repeat II	Repeat III
32	4	5	4	19	1	2	1
34	5	4	5	39	1	2	1
45	5	5	5	63	1	2	2
61	5	4	5	68	2	1	1
79	5	4	5	96	1	1	1
103	5	4	5	105	2	1	2
104	5	5	5	114	2	1	1
127	5	5	5	148	1	1	2
128	5	5	4	172	1	2	1
159	5	5	5	174	1	1	2
161	5	4	4	179	2	1	1
163	4	5	4	183	1	1	2
173	5	4	4	189	1	1	1
175	5	5	4	197	1	2	2
192	5	4	4	227	1	1	1
228	5	5	4	229	2	1	2
233	4	5	5	239	1	2	1
247	5	5	5	252	2	1	1
270	5	5	4	253	1	1	1
288	5	4	4	268	1	2	1
299	5	5	5	275	1	1	1
302	4	4	4	282	2	2	1
325	5	4	5	283	2	2	1
341	5	5	4	287	2	1	1
355	5	5	4	342	1	2	1
361	4	5	5	357	1	2	2
366	5	5	3	373	2	2	1
391	5	4	4	385	1	2	2
393	5	5	5	386	1	1	1
403	5	5	4	390	1	2	2

Variety	SBS-susceptible parent			Variety	SBS-resistant parent		
	Repeat I	Repeat II	Repeat III		Repeat I	Repeat II	Repeat III
ROC22	4	5	5	YT93-159	2	1	1

[a] represents the line number of the hybrid progeny in the F_1 population.
[b] SBS represents sugarcane brown stripe.
