# Supplementary material for: Screening of Candidate Genes Associated with Brown Stripe Resistance in Sugarcane via BSR-seq Analysis
Source: Int J Mol Sci. 2022 Dec 7;23(24):15500. doi: 10.3390/ijms232415500 (PMC9778799; doi:10.3390/ijms232415500)
Supplement: Supplementary file 1 [file ijms-23-15500-s001.zip › Supplementary_Material - Table S2.pdf]

International Journal of
Molecular Sciences

Supplementary Material

Table S2 Statistical table of associate region information filtered based on ED algorithm

Chromosome ID	Start	End	Size (Mb)	Gene number
Chr4B	68,425,062	68,425,062	0.000	1
Chr4B	68,719,509	68,719,509	0.000	1
Chr4B	68,792,592	69,647,491	0.850	39
Chr4B	69,676,447	69,676,447	0.000	1
Chr4B	70,470,674	71,011,619	0.540	24
Chr4B	71,877,891	76,876,764	5.000	194
Chr5C	82,284,273	82,495,391	0.210	10
Chr5C	85,651,974	89,339,122	3.690	102
Chr7C	10,498,147	10,529,260	0.030	2
Chr7C	29,243	8,032,576	8.000	348
Chr7C	8,197,136	8,338,936	0.140	8
Chr7C	8,754,945	8,754,945	0.001	1
Total	-	-	18.46	731
Non-synonymous mutant genes	-	-	-	24
