# Supplementary material for: Screening of Candidate Genes Associated with Brown Stripe Resistance in Sugarcane via BSR-seq Analysis
Source: Int J Mol Sci. 2022 Dec 7;23(24):15500. doi: 10.3390/ijms232415500 (PMC9778799; doi:10.3390/ijms232415500)
Supplement: Supplementary file 1 [file ijms-23-15500-s001.zip › Supplementary_Material - Table S3.pdf]

International Journal of
Molecular Sciences

Supplementary Material

Table S3 Statistical table of associate region information filtered based on SNP-index

Chromosome ID	Start	End	Size (Mb)	Gene number
Chr1D	114,687,846	114,687,846	0.000	1
Chr1D	114,929,083	114,929,083	0.000	1
Chr1D	115,107,002	115,107,002	0.000	1
Chr1D	25,103	2,537,258	2.510	114
Chr4B	69,647,638	69,709,690	0.060	7
Chr4B	70,264,523	71,013,990	0.750	35
Chr6D	76,683,768	76,695,084	0.010	4
Chr6D	77,624,276	77,628,095	0.000	2
Chr6D	82,160,511	82,323,181	0.160	7
Chr7C	29,243	14,360,553	14.33	580
Total	-	-	17.84	752
Non-synonymous mutant genes	-	-	-	28
