# Supplementary material for: Screening of Candidate Genes Associated with Brown Stripe Resistance in Sugarcane via BSR-seq Analysis
Source: Int J Mol Sci. 2022 Dec 7;23(24):15500. doi: 10.3390/ijms232415500 (PMC9778799; doi:10.3390/ijms232415500)
Supplement: Supplementary file 1 [file ijms-23-15500-s001.zip › Supplementary_Material - Table S4.pdf]

International Journal of
Molecular Sciences

Supplementary Material

Table S4 Localization of candidate regions of resistance to SBS based on ED algorithm and ΔSNP-index

Chromosome ID	Start	End	Size (Mb)	Gene number
Chr4B	69,676,447	69,676,447	0.00	1
Chr4B	70,470,674	71,011,619	0.54	24
Chr7C	10,498,147	10,529,260	0.03	2
Chr7C	29,243	8,032,576	8.00	348
Chr7C	8,197,136	8,338,936	0.15	8
Chr7C	8,754,945	8,754,945	0.00	1
Total	-	-	8.72	384
