# Supplementary material for: Screening of Candidate Genes Associated with Brown Stripe Resistance in Sugarcane via BSR-seq Analysis
Source: Int J Mol Sci. 2022 Dec 7;23(24):15500. doi: 10.3390/ijms232415500 (PMC9778799; doi:10.3390/ijms232415500)
Supplement: Supplementary file 1 [file ijms-23-15500-s001.zip › Supplementary_Material - Table S5.pdf]

International Journal of *Molecular Sciences*

Supplementary Material

Table S5 Gene functional annotation of the candidate genes

Candidate genes	Gene ID in *Arabidopsis*	Conserved domains	Gene description
Sspon.07G0005050-3C	AT3G57260.2	Glyco_hydro super family	Pathogenesis-related protein 2 (PR2)
Sspon.07G0002070-2C	AT3G23690.1	CIL2	CIB1 Like protein 2 (CBL2)
Sspon.07G0000630-2C	AT5G07070.1	SnRK3.2	CBL-interacting protein kinase 2 (CIPK2)
Sspon.07G0000430-3C	AT1G69270.1	PKc_like super family	Receptor-like protein kinase 1 (RLK1)
Sspon.07G0001660-2C	AT5G61580.1	PFK4	Phosphofructo kinase 4 (PFK4)
Sspon.07G0001100-1T	AT4G23650.1	STKc_CAMK	Calcium dependent protein kinase 6 (CDPK6)
Sspon.07G0003980-3C	AT3G44730.1	Motor_domain super family	Kinesin-like protein 1 (KP1)
Sspon.07G0030150-1C	AT4G24240.1	WRKY7	WRKY DNA-binding protein 7 (WRKY7)
Sspon.04G0018770-2B	AT1G61340.1	IPR001810	F-box stress induced 1 (FBS1)
Sspon.07G0001710-3C	AT3G02020.1	IPR042199	Aspartate kinase 3 (AK3)
Sspon.07G0021450-2C	AT5G67380.1	IPR011009	Casein kinase alpha 1 (CKA1)
Sspon.07G0001810-3C	AT2G23760.3	SBF super family	BEL1-like homeodomain protein 4 (BLH4)
Sspon.05G0036660-1C	AT2G47630.2	Abhydrolase super family	Monoacylglycerol lipase 9 (MAGL9)
Sspon.04G0020120-2B	AT2G40250.1	WRKY31	WRKY DNA-binding protein 31 (WRKY31)
Sspon.04G0020890-2P	AT5G14950.1	PLN02602	Golgi alpha-mannosidase II (GMII)
Sspon.05G0028560-1T	AT1G09970.1	PLN00113	Leucine-rich repeat receptor-like protein kinase (LRR-RLK)
Sspon.04G0029470-1B	AT3G12690.1	PR011009	AGC kinase 1.5 (AGC1.5)
Sspon.05G0036610-1C	AT3G47340.1	PLN02549 super family	Glutamine-dependent asparagine synthase 1 (ASN1)
Sspon.05G0021060-3C	AT1G08490.1	AAT_I super family	Chloroplastic NIFS-like cysteine desulfurase (CPNIFS)
Sspon.04G0019850-2B	AT5G65280.1	LANC-like	GCR2-like 1 (GCL1)
Sspon.07G0016690-1P	AT5G10240.1	PLN02549	Asparagine synthetase 3 (ASN3)
Sspon.04G0018310-2B	AT2G29990.1	Pyr_redox_2 super family	NAD(P)H dehydrogenase 2 (NDA2)

Candidate genes	Gene ID in Arabidopsis	Conserved domains	Gene description
Sspon.05G0036600-1C	AT4G19960.1	PLN02549 super family	K+ uptake permease 9 (KUP9)
Sspon.01G0037340-3D	AT1G09970.1	PLN02822	Receptor-like kinase 7 (RLK7)
Sspon.07G0007220-2C	AT1G36180.1	BRCT super family	Acetyl-CoA carboxylase 2 (ACC2)
Sspon.06G0027030-2D	AT4G11530.1	PKc_like super family	Cysteine-rich RLK (receptor-like protein kinase) (CRK34)
Sspon.01G0033610-2P	AT5G25830.1	DUF668	GATA transcription factor 12 (GATA12)
Sspon.01G0000580-4D	AT5G20610.1	ProQ super family	Plastid movement impaired1-related1 (PMIR1)
Sspon.07G0005710-2C	AT1G67120.1	MDN1 super family	Midasin 1 (MDN1)
Sspon.07G0006200-3C	AT3G09560.1	HAD_like	Phosphatidic acid phosphohydrolase 1 (PAH1)
Sspon.07G0030820-1C	AT2G18450.1	COG5028	Succinate dehydrogenase 1-2 (SDH1-2)
Sspon.07G0006660-3C	AT2G17780.1	Peptidase_C39_like	Mid1-complementing activity 2 (MCA2)
Sspon.07G0030530-1C	AT4G02600.1	NB-ARC domain	Mildew resistance locus O 1 (MLO1)
Sspon.01G0000980-1P	AT3G01540.1	F-box-like	Dead box RNA helicase 1 (DRH1)
Sspon.07G0005650-2C	AT5G43980.1	DUF1719	Plasmodesmata-located protein 1A (PDLP1A)
Sspon.04G0029090-1B	AT2G30360.1	DUF1645	CBL-interacting protein kinase 11 (CIPK11)
Sspon.07G0006670-3C	AT5G16710.1	Glutathione-S-Trfase_C	Dehydroascorbate reductase 1 (DHAR1)
Sspon.04G0018410-2B	AT2G17260.1	GST_C_family super family	Glutamate receptor 2 (GLR2)
Sspon.07G0001720-2C	AT5G46330.1	Leucine-rich repeat	Flagellin-sensitive 2 (FLS2)
