# Supplementary material for: Screening of Candidate Genes Associated with Brown Stripe Resistance in Sugarcane via BSR-seq Analysis
Source: Int J Mol Sci. 2022 Dec 7;23(24):15500. doi: 10.3390/ijms232415500 (PMC9778799; doi:10.3390/ijms232415500)
Supplement: Supplementary file 1 [file ijms-23-15500-s001.zip › Supplementary_Material - Table S6.pdf]

International Journal of
Molecular Sciences

Supplementary Material

Table S6 Statistical table of the number of differentially expressed genes in the candidate regions

DEG Set	DEG Number	Up-regulated	Down-regulated
T01 vs. T02	27	17	10
T03 vs. T04	22	5	17
Total	49	-	-

DEG Set, differentially expressed gene set name; DEG Number, number of differentially expressed genes; Up-regulated, the number of genes upregulated; Down-regulated, the number of genes downregulated.
