# Supplementary material for: Screening of Candidate Genes Associated with Brown Stripe Resistance in Sugarcane via BSR-seq Analysis
Source: Int J Mol Sci. 2022 Dec 7;23(24):15500. doi: 10.3390/ijms232415500 (PMC9778799; doi:10.3390/ijms232415500)
Supplement: Supplementary file 1 [file ijms-23-15500-s001.zip › Supplementary_Material - Table S8.pdf]

International Journal of Molecular Sciences

Supplementary Material

Table S8 Primers used in this study

Primer name	Forward primer	Reverse primer
GAPDH-Q	CACGGCCACTGGAAGCA	TCCTCAGGGTTCCTGATGCC
PR2-Q	CCGTCCAGTTCCGGTACATC	ACGACAGGTACGACACGATG
CBL2-Q	CTCTGCCTGAAGAGCGCATA	TCTTGTCCCCACCAGAGACA
CKA1-Q	GAAGCGCATCCTCATCGAGA	GGGCGGTCGATGATCAGATT
DHAR-Q	GCTCAAGCTGCAGAGGGAG	CTGTCTTCCTCTTCGGCTCC
CIPK11-Q	CGGCAAGGAGAAGTTCGTCT	TAGGGGAGGTACGACTGCTG
LRR-RLK-Q	TGGCCTACTCGCTCAAAGTG	TGAGCATCTTCACCACGTCC
NDA2-Q	TACAGGACATCAGGCGGAGA	CAGGTTCTTCGCCACTAGCA
WRKY31-Q	TGTGATCAACGTGCCACTGA	CAACTTACCAGCGGCATGTG
CDPK6-Q	AGATCATGCACCACCTCACG	TTGAGGTCCCGATGCATGAC
BLH4-Q	AGGACTCACCCTGCGAACTA	CAAAGACACCCCAGCTCCAG
GATA12-Q	GCAGCTGGTGTTCTCCAAGA	GGCGTATCTGATTCCGACCG
RLK7-Q	GTCTCGTCTGCGTTCAATGC	TATTCTGGCCCCCTCCTTGA
CIPK2-Q	AGCTTACTTCAGTGCCCGAC	TTTAGAACTGCTGGGCTCGG
WRKY7-Q	AAGAGTGATCCATCCACGGC	GCAGGCTTCAAATCAGCAGG
CRK34-Q	GCAGCTTGTCCATCATGTGC	GGTGTCGATGTGGCTGATGA
FLS2-Q	GCTCCCGTCATCCATATGCA	GCAGCACACCTTCGAGTTTG
PFK4-Q	AGGAGGCTGTGCTCAAGAAC	ATCCCCAATCTCCTCCTGCT
AK3-Q	GAGCTATGACATGGGGAGGC	TGCTAGCACAATGCCCAAGA
KUP9-Q	ACTGCCTGAGAGCCAACAAG	CTGTACCCAACGCCATCACT
RLK1-Q	CTGGTCAGGAGCAAGTCGAG	CCAATGGCCCCATCCAAGAT
