# Supplementary figures and images for: Screening of Candidate Genes Associated with Brown Stripe Resistance in Sugarcane via BSR-seq Analysis
Source: Int J Mol Sci. 2022 Dec 7;23(24):15500. doi: 10.3390/ijms232415500 (PMC9778799; doi:10.3390/ijms232415500)

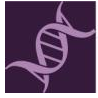

### Supplementary Material

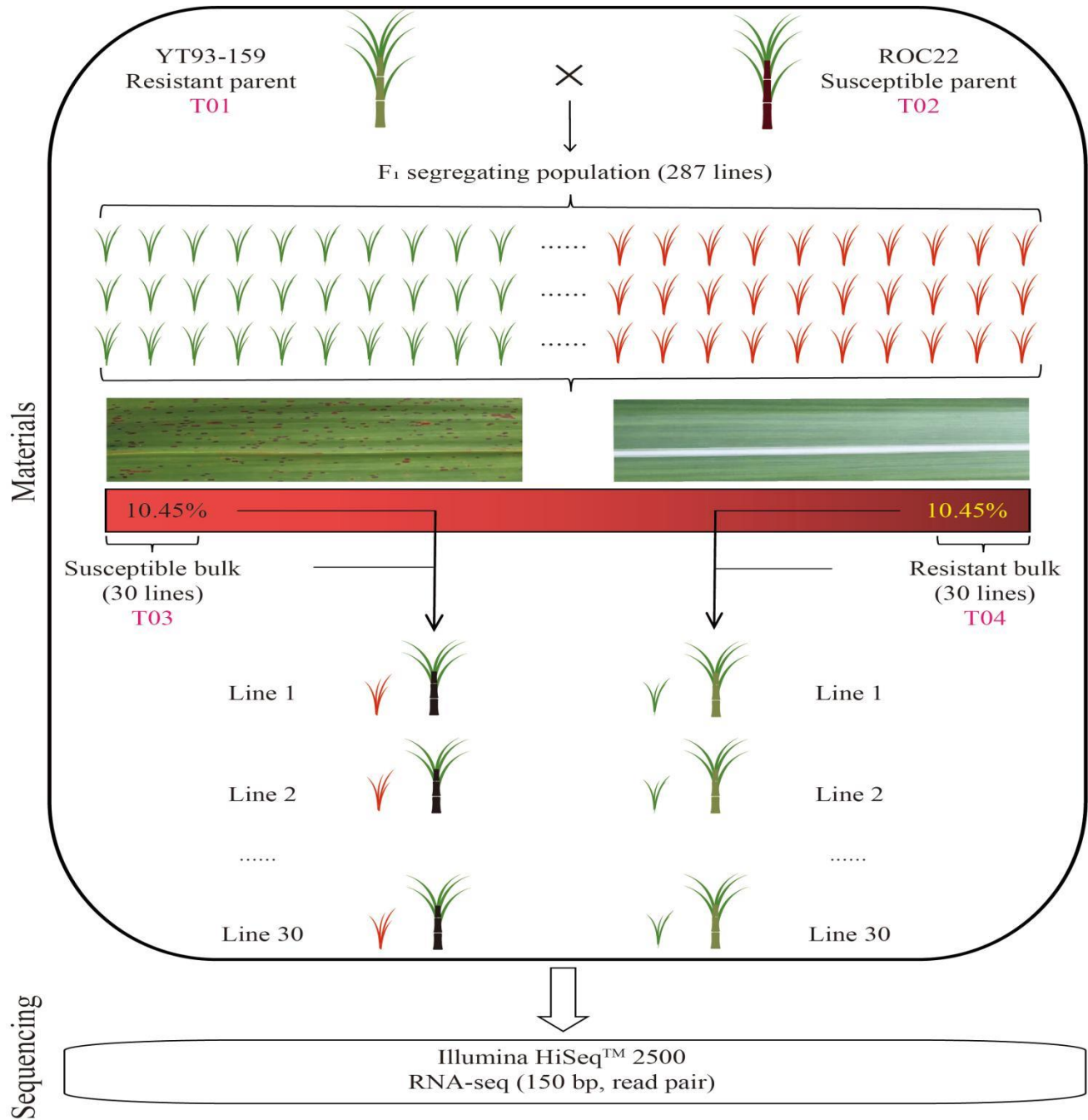

**Figure S4.** Schematic diagram of BSR-seq.

Supplement: Supplementary file 1 [file ijms-23-15500-s001.zip › Supplementary_Material - Figure S4.pdf]
